# Defining the extracellular matrix in non-cartilage soft-tissues in osteoarthritis: a systematic review

**DOI:** 10.1302/2046-3758.1312.BJR-2024-0020.R1

**Published:** 2024-12-03

**Authors:** Iwan G. A. Raza, Sarah J. B. Snelling, Jolet Y. Mimpen

**Affiliations:** 1 Medical Sciences Division, University of Oxford, Oxford, UK; 2 Botnar Institute for Musculoskeletal Sciences, Nuffield Department of Orthopaedics Rheumatology and Musculoskeletal Sciences, University of Oxford, Oxford, UK; 3 Kennedy Institute of Rheumatology, Nuffield Department of Orthopaedics Rheumatology and Musculoskeletal Sciences, University of Oxford, Oxford, UK

**Keywords:** Osteoarthritis, Extracellular matrix, Human, Animal models, Synovium, Meniscus, Osteoarthritis (OA), soft-tissues, cartilage, collagens, joint and tissue, meniscus, collagen fibres, Ligaments, tendons

## Abstract

**Aims:**

Extracellular matrix (ECM) is a critical determinant of tissue mechanobiology, yet remains poorly characterized in joint tissues beyond cartilage in osteoarthritis (OA). This review aimed to define the composition and architecture of non-cartilage soft joint tissue structural ECM in human OA, and to compare the changes observed in humans with those seen in animal models of the disease.

**Methods:**

A systematic search strategy, devised using relevant matrix, tissue, and disease nomenclature, was run through the MEDLINE, Embase, and Scopus databases. Demographic, clinical, and biological data were extracted from eligible studies. Bias analysis was performed.

**Results:**

A total of 161 studies were included, which covered capsule, ligaments, meniscus, skeletal muscle, synovium, and tendon in both humans and animals, and fat pad and intervertebral disc in humans only. These studies covered a wide variety of ECM features, including individual ECM components (i.e. collagens, proteoglycans, and glycoproteins), ECM architecture (i.e. collagen fibre organization and diameter), and viscoelastic properties (i.e. elastic and compressive modulus). Some ECM changes, notably calcification and the loss of collagen fibre organization, have been extensively studied across osteoarthritic tissues. However, most ECM features were only studied by one or a few papers in each tissue. When comparisons were possible, the results from animal experiments largely concurred with those from human studies, although some findings were contradictory.

**Conclusion:**

Changes in ECM composition and architecture occur throughout non-cartilage soft tissues in the osteoarthritic joint, but most of these remain poorly defined due to the low number of studies and lack of healthy comparator groups.

Cite this article: *Bone Joint Res* 2024;13(12):703–715.

## Article focus

- Extracellular matrix (ECM) is a critical determinant of tissue mechanobiology and cell behaviour, but it is poorly described in osteoarthritic joint tissues beyond cartilage.
- The main aim of this systematic review is to consolidate existing data describing the architecture and composition of structural ECM in the synovium, joint capsule, skeletal muscle, tendon, ligament, meniscus, intervertebral disc, and fat pad of osteoarthritic joints.

## Key messages

- Our study highlights the global nature of ECM dysregulation across the osteoarthritic joint.
- While some ECM changes, notably calcification and the loss of collagen fibre organization, have been extensively studied across osteoarthritic tissues, most ECM features were only studied by one or a few papers in each tissue.
- Results from animal studies generally concurred with human studies, but some findings contradicted observations from human studies, highlighting the importance of the choice of animal model and the need for validation from human studies.

## Strengths and limitations

- This systematic review consolidates existing knowledge of a poorly defined aspect of osteoarthritis pathophysiology.
- While a wide range of tissues and ECM components have been reported on, the qualitative nature of papers, the lack of control groups, and the paucity of reports on each ECM component means that the depth of knowledge remains poor.

## Introduction

Osteoarthritis (OA) is the most common joint disease globally, affecting over 500 million people. OA is typically attributed to mechanically driven joint damage and is characterized by articular cartilage degeneration and subchondral bone remodelling.^1^ However, these tissues are not affected in isolation from the wider joint, with pathology in other soft joint tissues contributing to the symptoms and progression of OA.^2,3^ Damage to menisci and ligaments disrupts joint biomechanics, while inflammation, fibrosis, and distension of the synovium and joint capsule are associated with joint pain and stiffness.^4-8^ Despite significant clinical need and substantial efforts to identify disease-modifying OA drugs, there is no effective way of inhibiting or decelerating OA-related joint damage by targeting cartilage directly. Given the important role of other soft-tissues in joint biomechanics and the release of pro-inflammatory and matrix-degrading mediators into the synovial fluid,^9,10^ understanding the biological landscape of the whole joint in OA might provide novel therapeutic strategies and prognostic markers.

Joint tissues are rich in extracellular matrix (ECM), a network of structural and regulatory macromolecules within which cells are embedded.^11^ The role of ECM as a major determinant of the biophysical properties of a tissue has clear relevance in a disease such as OA.^12,13^ ECM not only provides structure to the tissue, but can also affect cell function through receptor engagement, mechanical cues, and the sequestration of growth factors and cytokines.^14-17^ Significant crosstalk occurs between cells and matrix components, such that pathological ECM may exacerbate cellular dysfunction in disease.^16,18^ Therefore, ECM composition and architecture cannot be disregarded when attempting to understand OA pathophysiology. However, outside of cartilage, ECM remodelling in OA tissues has received relatively little attention.

Studying OA in the clinical setting is challenging due to the slow and unpredictable nature of the course of the disease. In addition, clinical symptoms often appear late in the disease process, making it difficult to study its onset and early progression. Therefore, many animal models for OA have been developed to overcome these issues and facilitate the development and evaluation of new therapies and diagnostic tools.^19^ However, since there is no single “gold standard” animal model that accurately reflects all aspects of human disease, a major challenge is selecting the “right” model for each study.^20^

The main aim of this systematic review is to consolidate existing data describing the architecture and composition of structural ECM in the synovium, joint capsule, skeletal muscle, tendon, ligament, meniscus, intervertebral disc, and fat pad of osteoarthritic joints. The second aim is to define the changes in the architecture and composition of structural ECM in these tissues in animal models of OA, in order to address their ability to replicate human disease pathophysiology.

## Methods

### Systematic review protocol and registration number

This review was conducted according to a protocol registered on the PROSPERO database (CRD42021231241) and guidelines set out in the PRISMA statement.^21^

### Database and search strategy

The search strategy, written by JYM and a medical librarian, can be found in the Supplementary Material. ECM components and architectural features were defined using National Centre for Biotechnology Information Medical Subject Heading terms.^22^ Non-cartilage soft joint tissues and disease nomenclature were also specified. The search strategy was validated against relevant papers identified in a preliminary literature search. The search strategy was run on the Ovid MEDLINE, Ovid EMBASE, and Scopus platforms on 30 October 2020 and repeated on 1 October 2021 and 1 June 2023.

### Eligibility criteria and screening

Abstracts were de-duplicated in Mendeley Reference Manager (Elsevier B.V., Netherlands) before being imported into the Covidence platform. The remaining studies were screened independently at title/abstract and full-text stages by two reviewers (JYM, IGAR), with conflicts resolves through consensus or a third reviewer (SJBS). Included studies were required to have ≥ three OA participants.

In human studies, eligible patients and controls were aged ≥ 18 years. Non-OA diseases, including inflammatory arthritides and crystalline arthropathies, were excluded. The presence of a valid control group was not a requirement for human studies. However, control groups were included if present and a minimum of three participants were included in this group. Valid control groups included tissues from healthy people or near-healthy tissues, including cadavers, individuals with osteosarcoma, and traumatic joint injuries provided that the comparator tissue was not directly damaged by the trauma.

In contrast to human studies, all animal studies required a control group. Studies that induced OA unilaterally and only used a contralateral control joint were excluded, as non-physiological loading of the contralateral joint induces ECM remodelling.^23,24^ Excluded animal models included the genetic deletion of ECM components, the introduction of matrix-degrading enzymes into the joint, surgical damage of a tissue subsequently reported on, and the ovariectomized rat model, as this is more commonly used as a model for osteoporosis.^25,26^

Regarding outcome measures, included studies evaluated at least one of the following tissues: intervertebral disc, ligament, skeletal muscle, tendon, meniscus, articular capsule, synovium, and fat pad. Papers that only studied these tissues after treatment, including – but not limited to – surgical or drug treatment, or after these tissues were purposely injured to induce the development of OA, were excluded. Papers evaluating non-ECM tissue components (cells, cytokines, matrix-degrading enzymes) were ineligible for inclusion. Given the focus on structural ECM, regulatory matricellular proteins, as well as neoepitopes generated during ECM turnover, were not included. Studies using in vitro or ex vivo culture systems were excluded as the ECM proteins that cells synthesize differ in culture and in vivo. Transcriptomic analyses were excluded as gene expression is a determinant, not a measure, of protein abundance. Finally, only English-language articles were included.

### Data extraction and bias analysis

Data were extracted from all included studies by one reviewer (JYM or IGAR) using a standardized extraction form in Microsoft Excel (Microsoft, USA); the extraction was verified by the other reviewer (IGAR or JYM). Where there was uncertainty, extraction was performed in duplicate by both reviewers. Number of participants (or animals) in each group was recorded as well as the presence/absence of a control group; if a control group was present, the control population and control tissue were described. For animal studies, the species, strain, and type of OA model were recorded. When available, participant age, sex, BMI, and disease severity were recorded, as were the joint and tissue being studied. Relevant ECM components and architectural features were described; comparisons to control tissues and statistical analysis were noted when applicable. Results were grouped by tissue, followed by ECM feature, and finally the direction of change compared to control (increase, no change, decrease, or no control group present) and presented in Supplementary Table i (human studies) and Supplementary Table ii (animal studies). Due to the large number of different included ECM features, accepted research methods, and accepted measures of effect, a quantitative meta-analysis was not deemed appropriate. Bias analysis was performed by IGAR, with all included studies assessed using the 2015 Office of Health Assessment and Translation (OHAT) Risk of Bias Rating Tool for Human and Animal Studies. The results of the bias analysis can be found in Supplementary Table iii.

## Results

### Study overview

**Fig. 1:**
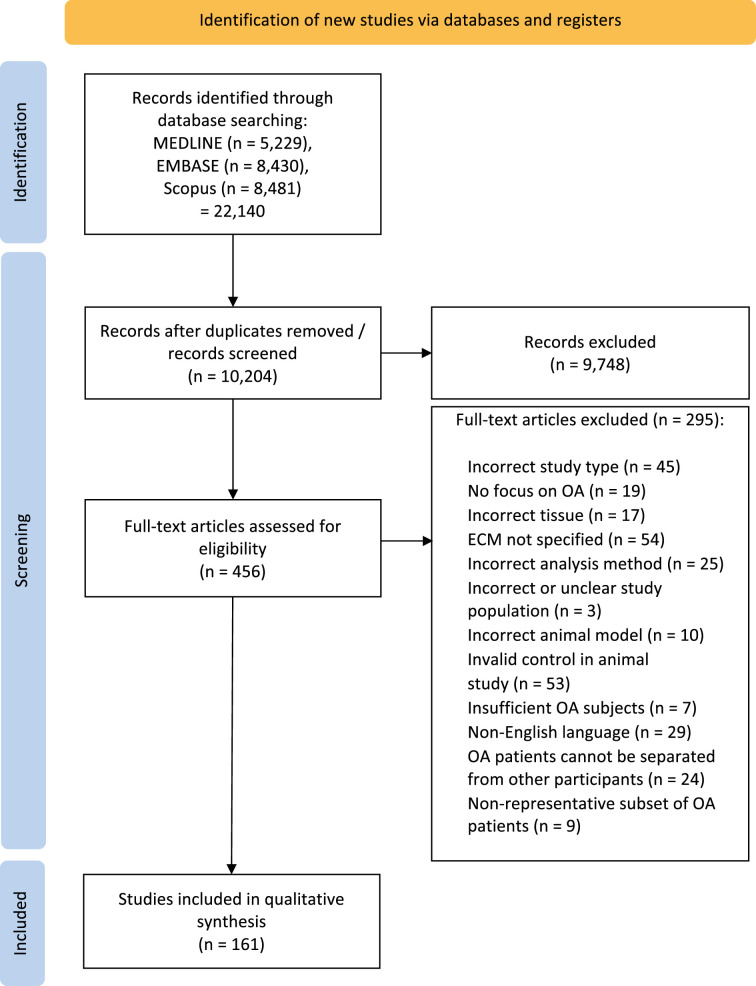
PRISMA 2022 flow diagram. ECM, extracellular matrix; OA, osteoarthritis.

**Fig. 2:**
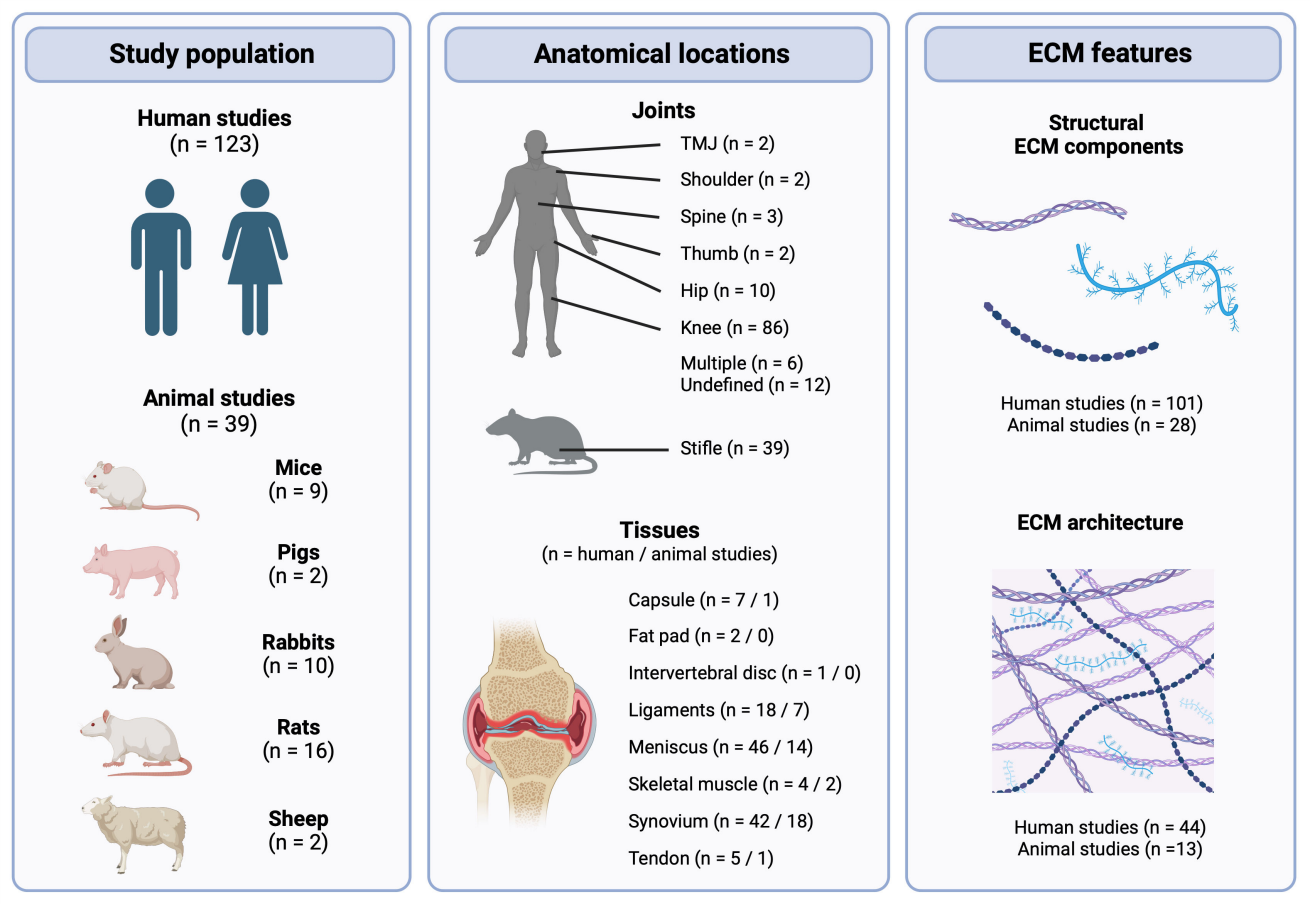
Schematic overview of the study population, anatomical locations, and extracellular matrix (ECM) features studied in the included studies. One study investigated ECM in both human osteoarthritis (OA) and an animal model of OA. Created with BioRender.com. TMJ, temporomandibular joint.

A total of 22,140 potentially relevant articles were identified by the search strategy (Figure 1). Following the removal of duplicates, 10,204 abstracts were screened. Of the 456 studies assessed for eligibility at full-text screening, 161 met all criteria for inclusion in this review. The characteristics of all included studies are summarized in Supplementary Tables iv and v (human and animal studies, respectively). A schematic overview of the included studies can be found in Figure 2.

### Human studies

Most studies investigated meniscus (n = 46) and synovium (n = 42), followed by ligaments (n = 18), capsule (n = 7), tendon (n = 5), skeletal muscle (n = 4), fat pad (n = 2), and intervertebral disc (n = 1) (Supplementary Table i). Studies most commonly investigated the knee joint (n = 86), but papers on hip (n = 10), spine (n = 3), thumb (n = 2), temporomandibular joint (TMJ) (n = 2), and shoulder (n = 2) were also identified. While most studies on synovium, tendon, and capsule focused on the presence/absence and distribution of specific ECM components, a large proportion of the papers on meniscus and ligaments investigated ECM architecture and viscoelastic properties (Supplementary Table i).

### Capsule in human OA

Of seven studies which assessed the capsule (hip (n = 3), knee (n = 3), and spine (n = 1)),^27-33^ four were published before the year 2000. These studies covered both ECM components and architectural features, but only collagen content was covered by more than one study, with two papers describing increased collagen staining.^28,29^ Voelker et al^30^ looked at several ECM components, showing an increase in type I collagen and no difference in type III collagen and elastin in OA facet joint capsule compared to cadaver controls.^30^ Of note, DiFrancesco et al^27^ studied several ECM features (calcification, collagen fibre organization, elastic fibres, and GAG/proteoglycan content) in parallel,^27^ providing an overview of hip capsule in OA. Other studies showed decreased collagen fibre organization,^32^ the presence of several GAGs,^33^ and an increase in collagen cross-links in OA.^31^

### Fat pad in human OA

Two studies were identified for infrapatellar fat pad.^34,35^ Grevenstein et al^35^ found no change in cartilage oligomeric matrix protein (COMP) content between OA and control fat pads,^35^ while Belluzzi et al^34^ showed that the osteoarthritic fat pad contains less collagen type I and III than controls.

### Intervertebral disc in human OA

One study was identified for intervertebral disc. Cheng et al^36^ showed an increase in calcification with increasing OA grade in intervertebral discs.

### Ligaments in human OA

Of the 18 studies on ligaments, 14 focused on anterior cruciate ligament (ACL) and/or posterior cruciate ligament (PCL) of the knee.^37-50^ Two studies looked at ligaments in the thumb (palmar beak ligament,^51^ volar anterior oblique (AOL), and dorsoradial (DRL)^52^), while two other studies investigated ligaments in the spine (transverse ligament^53^ and the ligamentum flavum).^30^ Studies mostly focused on collagen fibre organization, which generally decreased in OA compared to control.^42-44^ Studies without controls also reported disorganized and irregular collagen fibre organization in OA ligaments. Other identified studies confirmed the presence of collagens I, II, and III, but found no change in overall collagen content compared to control. In contrast, calcification and proteoglycan content appear to increase in OA.

### Meniscus in human OA

Studies on human meniscus (n = 46) covered a wide range of ECM components, architectural changes, and viscoelastic properties.^54-99^ Most studies concur on an increase in calcification and proteoglycan content, and consistently show a decrease in collagen fibre diameter and organization. The presence or change in many other ECM components has been studied, including aggrecan, biglycan, cartilage intermediate layer protein, collagens and collagen cross-links, COMP, decorin, fibromodulin, glycosaminoglycan (GAG) components, hydroxyproline, keratocan, lubricin, and lumican. Notably, three out of four proteomics studies included in this systematic review evaluated human OA meniscus, identifying a range of ECM and ECM-associated proteins.^97-99^ Two of these studies (Folkesson et al^97^ and Roller et al^98^) also analyzed control samples and found several proteins to be changed in OA compared to control tissue. For example, both studies report an increase in type VI α 1 collagen and type VI α 2 collagen in OA, and Folkesson et al^97^ found a change in protein abundance in several small leucin-rich proteoglycans, such as an increase in lumican and decrease in decorin, an increase in the proteoglycans aggrecan and versican, and a decrease in type III and V collagens.^97,98^ Finally, the results on viscoelastic properties are conflicting: while some studies show an increase in elastic modulus^89^ and instantaneous modulus,^90^ another study showed a decrease in these parameters.^61^

### Skeletal muscle in human OA

All four studies on human skeletal muscle studied the ECM components in the vastus medialis or vastus lateralis of the quadriceps muscle.^100-103^ These studies demonstrated the presence^103^ or increase^102^ in type I, III, and IV collagens compared to control. In addition, these studies show the presence of calcification and laminin,^100,102^ and an increase in collagen and GAG content.^101^

### Synovium in human OA

Synovial tissue was studied in several joints, including the knee (n = 18),^94,104-120^ hip (n = 5),^121-125^ both knee and hip (n = 6),^126-131^ TMJ (n = 2),^132,133^ or an unspecified joint (n = 12).^130,134-144^ The ECM components most often studied in human synovium were collagens, fibronectins, and laminins. Other ECM features covered by the included studies are aggrecan, calcification, collagen content, collagen fibre organization, collagen cross-links, COMP, elastin, fibromodulin, GAG components, latent transforming growth factor (TGF)-β-binding protein 1, lumican, reticulin, and vitronectin. While the presence and tissue distribution of these components has been clearly shown by several studies, the changes between OA and normal tissue remain unclear, with most studies lacking healthy control groups; instead, OA is often the comparator group in studies investigating rheumatoid arthritis (RA). This includes the identified proteomics study, which compared the OA and RA synovium. They found that several ECM proteins, including type 2 α 1 collagen, versican, and cartilage intermediate layer protein 1 were higher in OA than RA synovium.^143^

### Tendon in human OA

Human tendon studies covered a range of different tendons across the body, including Achilles, (long head of) biceps, subscapularis, gluteus medius, and internal obturator.^145-149^ Discordant results between studies of anatomically distinct tendons are unsurprising, but disagreement was also seen for two studies on biceps tendon. For example, GAG/proteoglycan content was increased in the long head of biceps and internal obturator tendon,^145,148^ unchanged in another study on biceps tendon and subscapularis tendon,^146^ and decreased in gluteus medius tendon in OA compared to control.^149^ Similarly, increased calcification was seen in obturator tendon,^145^ while there was no difference in subscapularis, and a decrease in biceps tendon.^146^ In terms of architecture, three out of four studies reporting on collagen fibre organization report a decrease in organization,^145,146,149^ while the last reported no difference compared to control.^148^ An increase in collagen fibre diameter was found in internal obturator and biceps tendon,^145,146^ while no difference was seen in subscapularis and gluteus medius tendons.^146,149^ Finally, no difference was found in the percentage area stained for type I and II collagen and decorin.^148^

### Animal studies

Animal studies followed a similar pattern as human studies regarding the most studied tissues: synovium (n = 18), meniscus (n = 14), ligament (n = 7), skeletal muscle (n = 2), tendon (n = 1), and capsule (n = 1) (Supplementary Table ii). A broad range of species, strains, and models were used, all looking at the stifle joint of these animals. Overall, these studies generally found increases in ECM components such as collagen and disrupted ECM architecture, including a decrease in collagen fibre organization in most tissues (Supplementary Table ii). Viscoelastic properties were mainly studied in meniscus, where the elastic and instantaneous modulus tended to decrease.

### Capsule in animal models of OA

Only one study was identified on capsule. Loeser et al^150^ studied capsule in the DMM model in C57BL/6 mice.^150^ Type III collagen was found to be diffusely expressed in OA capsule, predominantly in vascular endothelium. Interestingly, this study also assessed the meniscus, ligament, and synovium, taking a whole-joint approach to OA; they report a diffuse distribution of type III collagen similar to capsule in ligaments and synovium, while there was a pericellular distribution in meniscus.

### Ligament in animal models of OA

Ligaments were studied in OA models in mice (n = 4),^150-153^ rabbits (n = 2),^154,155^ and sheep (n = 2).^156,157^ A decrease in collagen fibre organization was reported by two studies.^155,157^ While one study reported an increase in GAG staining using toluidine blue in ACL of STR/ort mice,^151^ another showed a decrease in Raman spectroscopy peaks related to GAG content in MCL/LCL of ACL transection (ACLT) rabbits.^154^ All other reported ECM features were only present in one study. These features include calcification, mineralization, collagen content, types II and III collagen, collagen cross-links, collagen fibre diameter, and mechanical strength.

### Meniscus in animal models of OA

ECM changes in meniscus in animal models of OA were investigated by six studies using mouse models,^150,151,158-161^ five studies using rabbit models,^162-166^ one study using a rat model,^167^ and two studies using a pig model.^168,169^ Overall, these studies show an increase in calcification/mineralization and types I, II, III, and X collagen, and a decrease in collagen fibre organization. Most studies show a decrease in GAG/proteoglycan content and viscoelastic properties in at least parts of the meniscus. In addition, thickening of the collagen fibres and no change in fibromodulin were found.

### Skeletal muscle in animal models of OA

Two studies were identified that investigated skeletal muscle. Shi et al^170^ studied the elastic modulus in biceps femoris and rectus femoris muscles in an adapted Videman method in rabbits; they report an increase in elastic modulus in OA compared to control.^170^ Lee et al^171^ investigated the rectus femoris muscle using a monoiodoacetate (MIA) model in rats; they reported a decrease in collagen levels on days 56 and 87 in OA rats compared to the naïve group.^171^

### Synovium in animal models of OA

Synovium was investigated in three studies using mouse models,^150,158,172^ 13 studies using rat models,^104,173-184^ and two studies using rabbit models.^185,186^ All studies on calcification, collagen content, and collagen I showed an increase in OA compared to control. However, results on collagen fibre organization and collagen fibre diameter were less clear, with some studies reporting no change, while others reported a decrease in collagen fibre organization and increase in collagen fibre diameter. Other studied features included types III, V, and XIV collagen, COMP, fibromodulin, lubricin, and viscoelastic properties (elastic modulus), which were each reported on by a single study.

### Tendon in animal models of OA

Tendon was investigated in one study by McErlain et al^187^ using an ACLT model in rats. They found calcification of the patellar tendon to be more common in OA than control animals.^187^

### Bias analysis

The risk of bias varied between studies but was generally high (Supplementary Table iii). The potential for confounding bias was common, with many human studies failing to report on the age, sex, and BMI of participants. Frequently, OA diagnoses were stated without reference to the diagnostic criteria used. Most studies failed to report on the blinding of assessors, even when qualitative histological observations were made. Purely qualitative observations were common, although semiquantitative scoring systems were increasingly used in more recent studies. However, many quantitative and semiquantitative differences between healthy and osteoarthritic tissues were not statistically analyzed.

## Discussion

Despite OA becoming more widely accepted as a whole joint disease, the role of and the changes to non-cartilage soft joint tissues remain underexplored. This study aimed to collate current knowledge on the structural ECM of these tissues to summarize and highlight gaps in existing knowledge. For instance, tissues such as the joint capsule and fat pad are very poorly defined, perhaps reflecting their perceived importance in OA. Overall, the studies included in this review show that the presence and/or abundance of many structural ECM components changes in disease, within an ECM that becomes less organized with increasing cartilage damage or increasing tissue-specific degeneration scores.

Human studies covered a range of tissues and ECM features, but focused mainly on calcification, the presence and abundance of proteoglycans, and the presence, abundance, fibre diameter, and fibre organization of collagens. While recent studies begin to define the presence and distribution of many ECM components, a frequent absence of well-defined control groups limits our understanding of the changes in disease. Most ECM features are only described by one or a few studies, highlighting the need for studies that cover multiple ECM features. While studies that did look at the same ECM feature mostly agreed, this was not always the case. This included studies with control groups that investigated the collagen content in meniscus,^54,72^ elastic modulus in meniscus,^61,89^ chondroitin sulphate in synovium,^119,130^ and calcification and GAG/proteoglycan content in tendon,^145,146,148,149^ which all contradict each other in terms of the direction of change. The summary and results tables highlight several potential factors for these differences already, including differences in analysis methods, tissue joint origin, and microanatomical area of studied tissue, emphasizing the importance of in-depth reporting of tissue metadata and methods.

Several recent human studies, mostly in ligaments, tendon, and meniscus, have begun to interrogate both compositional and architectural ECM features within a single tissue. Importantly, such studies can begin to dissect the relationship, including causality, between changes in ECM composition, ECM architecture, and viscoelastic properties. For example, studies in the field have shown that calcification of tendon changes its viscoelastic properties,^188^ while the mechanical properties of fibril-forming collagens are dependent on covalent cross-linking,^189^ and different matrix proteoglycans differ in their effects on cell-mediated collagen reorganization.^190^

Whole tissue proteomics, which can be used to study the ECM composition of a tissue holistically, was performed in four studies: three on meniscus^97-99^ and one on synovium.^143^ While the study of ECM proteins using proteomic techniques is subject to methodological biases due to their large size, extensive post-translational modification, and insolubility,^191^ they are a powerful tool to better understand relative abundance of ECM proteins and overall tissue composition and formulate new research questions. The application of this technique to other osteoarthritic tissues is likely to provide important insights.

In animal models, OA is induced in a range of species using varied surgical techniques and pharmacological interventions, with no animal model truly replicating human disease.^19,192^ Joint mechanics, inflammatory responses, and disease chronicity all vary between animal models.^192,193^ If ECM remodelling also differs between species and procedures, it can be assumed that not all animal models are equally suited to the study of changes in osteoarthritic ECM. Certain models may be generally more representative of changes seen in human OA, or better suited to the study of particular joint tissues or ECM features. This review covers a range of ECM changes in several different musculoskeletal soft-tissues across different species and models. Although limited animal studies were eligible for inclusion in this review, some changes in ECM features could be compared between human OA and animal models. Generally similar trends could be seen as in humans, including a decrease in collagen fibre organization and an increase in calcification across ligaments, meniscus, and synovium. However, other observations seem to contradict those in humans; for example, the presence and abundance of collagens seemed to decrease in human osteoarthritic menisci, especially with increasing degeneration of the meniscus,^54,73,75^ while this is not reflected in data from any of the animal models in this review, which mainly showed increases in collagens in OA menisci.^151,161,163^ Therefore, the models used by these studies, namely the mouse STR/ort, rabbit ACLT, and mouse DMM models, respectively, might not be suitable to infer OA-related changes in human menisci. These results emphasize that more studies on ECM changes in non-cartilage soft joint tissues in human OA and animal models must be compared before the validity of the latter can be accurately defined.

Another important point to note is the difference in the ratio of female/male subjects in human studies compared to this ratio in animal studies: while most human studies include a higher ratio of female than male subjects, many animal studies are done exclusively using male animals. The predominance of women in human studies likely reflects disease prevalence; sex-specific differences in pain, inflammation, cartilage volume, and physical difficulty exist in OA,^194^ as well as in the presence of risk factors for the incidence of radiological knee OA.^195^ The presence of a sex bias in preclinical research is well established, with many fields having a strong male bias during animal studies.^196^ Encouragingly, sex-specific differences in animal models of OA are increasingly being addressed and reported on, including differences in the progression of the disease and response to pain.^197-201^ This emphasizes the importance of accounting for sex during the interpretation of results from both human and animal research studies to the human OA patient population.

The strength of any systematic review is partly contingent on the quality of included studies. As discussed in the Results section on bias analysis, the methodology of many studies conferred a high risk of bias, resulting in a low confidence in the evidence provided. In basic science studies utilizing human samples, the baseline characteristics and clinical characterization of OA patients are often missing, or lack necessary detail. Clinical background is a particularly important consideration in the context of soft-tissue calcification, given that crystal depositional diseases, such as pseudogout, can drive OA.^202^ Patients’ clinical background is poorly reported throughout the literature, as is disease severity, despite ECM and other tissue components differing more from the physiological state with OA progression.^42^ As clinical information might not always be available for collection due to ethical constraints, making this clear to readers allows findings to be interpreted in the correct clinical context. Although the search strategy covered many non-cartilage soft joint tissues, some tissues, such as the temporomandibular joint disc and acetabular labrum, were not included. In addition, the focus of this review was on structural components of the ECM, which are the elements that are studied most extensively and make up the majority of tissue ECM. However, this does mean that this work does not provide a complete account of all OA ECM, as non-structural matrix elements such as matricellular proteins or neoepitopes have not been reported on. Finally, a limitation of the review process is the data extraction, which was not done by two independent reviewers, but rather extracted by one reviewer and verified by the other reviewer. However, the effect of this is likely limited as a previous study has reported that while extraction by two independent reviewers is preferable, extraction by one reviewer with verification by a second reviewer has limited influence on the conclusions of a systematic review, especially considering a meta-analysis was not performed in the current work.^203^

In the process of consolidating the current literature on this topic, this work highlights several practical and methodological challenges that have limited progress in the understanding of structural ECM components, architectural features, and viscoelastic properties in non-cartilage soft-tissues in OA. One of these problems is the cross-sectional nature of studies, which is popular in the OA field as tissues are only accessible at the time of joint arthroplasty. Since OA can take decades to progress, the study of end-stage or advanced OA might not be fully informative of the processes that are driving these changes. In addition, the lack of a healthy, or non-OA, comparator group, in combination with the fact that many studies only report qualitative results, vastly reduces the depth of knowledge that can be gained from these studies. Finally, while many screened human and animal studies investigated both cartilage and other soft joint tissues, ECM is often studied exclusively in cartilage, with other features, such as cellularity and inflammatory markers, being the focus in other tissues. This shows that while there is access to both the tissues and the methods to study ECM changes in non-cartilage soft-tissues, the analysis of these tissues is not seen as a priority. However, due to the limited characterization of ECM in these tissues and their unknown contribution to disease development and progression, it is also possible that it remains unclear which ECM feature(s) should be focused on. Structural ECM encompasses a wide range of features that can be investigated with a plethora of different methods. To evaluate the most critical ECM features and applicable methods, studies investigating multiple ECM features in non-cartilage soft-tissues across different stages of disease are required.

Recent studies have started to highlight the importance of ECM as a determinant of tissue architecture and cell behaviour in disease. For example, a recent review highlights that the changes in microenvironment in early RA form important extracellular cues that shape the pathogenic cell behaviour during the onset and progression of disease.^204^ Therefore, the authors argue that understanding the ECM changes across different tissues in a particular disease might not only be able to help with disease classification and patient stratification, but could also hold promise for the development of treatments that target ECM.^204^ These treatments might not only be able to modify pathogenic cell behaviour that could be driving the disease, but also impact on joint stiffness, which is one of the most common symptoms of OA.^205^ All in all, more research is needed to unravel the presence and distribution of different ECM components and architectural features in joint tissues in health and in (different stages of) OA, and interplay with tissue-resident and tissue-infiltrating cells. Future research will also help to differentiate between the remodelling process in different joint tissues, which contain unique cell populations and are exposed to different mechanical and inflammatory stimuli in OA. ECM remodelling may also differ between synovial joints, given their varied anatomical locations, mechanical functions, and the presence of joint-specific tissues such as menisci. Potential variation in pathophysiology between OA joints has received little attention, with the predominance of studies on knee OA likely due to high disease prevalence in this joint and tissue being relatively accessible during commonly performed knee arthroplasties. Therefore, the future of this field is both dependent on the thorough investigation of ECM features in non-cartilage soft joint tissues across multiple OA joints and varied stages of disease progression, as well as the rigorous reporting of patient characteristics of all tissue donors.

In conclusion, this systematic review consolidates existing knowledge of a poorly defined aspect of OA pathophysiology. While a wide range of tissues and ECM components have been reported on, the qualitative nature of papers, the lack of control groups, and the paucity of reports on each ECM component means that the depth of knowledge remains poor. Overall, the studies included in this review show that the presence and abundance of many structural ECM components change in OA, and that the ECM architecture becomes more disorganized with increasing cartilage damage or increasing tissue-specific degeneration scores. While results from animal studies generally concurred with human studies, some findings contradicted observations from human studies, highlighting the importance of the choice of animal model and the need for validation in human studies. Given the role of ECM in influencing cell behaviour, further research to elucidate the broad context within which cartilage is damaged in OA will provide more insight into the disease as well as potential treatments.

## Data Availability

The authors confirm that the data supporting the findings of this study are available within the article and its supplementary materials. In addition, the raw data from the data extraction process, which were used to populate Supplementary Tables i, ii, iv, and v, are available upon reasonable request from the corresponding author.

## References

[1] Goldring SR Goldring MB Changes in the osteochondral unit during osteoarthritis: structure, function and cartilage-bone crosstalk Nat Rev Rheumatol 2016 12 11 632 644 10.1038/nrrheum.2016.148 27652499

[2] Mimpen JY Snelling SJB Chondroprotective factors in osteoarthritis: a joint affair Curr Rheumatol Rep 2019 21 8 41 10.1007/s11926-019-0840-y 31227927 PMC6588640

[3] Poole AR Osteoarthritis as a whole joint disease HSS J 2012 8 1 4 6 10.1007/s11420-011-9248-6 23372516 PMC3295952

[4] Zhang K Li L Yang L et al. The biomechanical changes of load distribution with longitudinal tears of meniscal horns on knee joint: a finite element analysis J Orthop Surg Res 2019 14 1 237 10.1186/s13018-019-1255-1 31345248 PMC6659249

[5] Shirazi R Shirazi-Adl A Analysis of partial meniscectomy and ACL reconstruction in knee joint biomechanics under a combined loading Clin Biomech (Bristol, Avon) 2009 24 9 755 761 10.1016/j.clinbiomech.2009.07.005 19656595

[6] Wellsandt E Gardinier ES Manal K Axe MJ Buchanan TS Snyder-Mackler L Decreased knee joint loading associated with early knee osteoarthritis after anterior cruciate ligament injury Am J Sports Med 2016 44 1 143 151 10.1177/0363546515608475 26493337 PMC4703470

[7] Hill CL Hunter DJ Niu J et al. Synovitis detected on magnetic resonance imaging and its relation to pain and cartilage loss in knee osteoarthritis Ann Rheum Dis 2007 66 12 1599 1603 10.1136/ard.2006.067470 17491096 PMC2095318

[8] Hill CL Gale DG Chaisson CE et al. Knee effusions, popliteal cysts, and synovial thickening: association with knee pain in osteoarthritis J Rheumatol 2001 28 6 1330 1337 11409127

[9] Sanchez-Lopez E Coras R Torres A Lane NE Guma M Synovial inflammation in osteoarthritis progression Nat Rev Rheumatol 2022 18 5 258 275 10.1038/s41584-022-00749-9 35165404 PMC9050956

[10] Wang M Tan G Jiang H et al. Molecular crosstalk between articular cartilage, meniscus, synovium, and subchondral bone in osteoarthritis Bone Joint Res 2022 11 12 862 872 10.1302/2046-3758.1112.BJR-2022-0215.R1 36464496 PMC9792876

[11] Theocharis AD Skandalis SS Gialeli C Karamanos NK Extracellular matrix structure Adv Drug Deliv Rev 2016 97 4 27 10.1016/j.addr.2015.11.001 26562801

[12] Urbanczyk M Layland SL Schenke-Layland K The role of extracellular matrix in biomechanics and its impact on bioengineering of cells and 3D tissues Matrix Biol 2020 85–86 1 14 10.1016/j.matbio.2019.11.005 31805360

[13] Felson DT Osteoarthritis as a disease of mechanics Osteoarthritis Cartilage 2013 21 1 10 15 10.1016/j.joca.2012.09.012 23041436 PMC3538894

[14] Klees RF Salasznyk RM Kingsley K Williams WA Boskey A Plopper GE Laminin-5 induces osteogenic gene expression in human mesenchymal stem cells through an ERK-dependent pathway Mol Biol Cell 2005 16 2 881 890 10.1091/mbc.e04-08-0695 15574877 PMC545919

[15] Du J Zu Y Li J et al. Extracellular matrix stiffness dictates Wnt expression through integrin pathway Sci Rep 2016 6 1 20395 10.1038/srep20395 26854061 PMC4745056

[16] Allen JL Cooke ME Alliston T ECM stiffness primes the TGFβ pathway to promote chondrocyte differentiation Mol Biol Cell 2012 23 18 3731 3742 10.1091/mbc.E12-03-0172 22833566 PMC3442419

[17] Wijelath ES Rahman S Namekata M et al. Heparin-II domain of fibronectin is a vascular endothelial growth factor-binding domain Circ Res 2006 99 8 853 860 10.1161/01.RES.0000246849.17887.66 17008606 PMC3175430

[18] Thomas CM Murray R Sharif M Chondrocyte apoptosis determined by caspase-3 expression varies with fibronectin distribution in equine articular cartilage Int J Rheum Dis 2011 14 3 290 297 10.1111/j.1756-185X.2011.01627.x 21816026

[19] McCoy AM Animal models of osteoarthritis: comparisons and key considerations Vet Pathol 2015 52 5 803 818 10.1177/0300985815588611 26063173

[20] Teeple E Jay GD Elsaid KA Fleming BC Animal models of osteoarthritis: challenges of model selection and analysis AAPS J 2013 15 2 438 446 10.1208/s12248-013-9454-x 23329424 PMC3675748

[21] Page MJ McKenzie JE Bossuyt PM et al. The PRISMA 2020 statement: an updated guideline for reporting systematic reviews BMJ 2021 372 71 10.1136/bmj.n71 33782057 PMC8005924

[22] No authors listed National Library of Medicine https://www.nlm.nih.gov/mesh/meshhome.html date last accessed 28 October 2024

[23] Poulet B de Souza R Kent AV et al. Intermittent applied mechanical loading induces subchondral bone thickening that may be intensified locally by contiguous articular cartilage lesions Osteoarthritis Cartilage 2015 23 6 940 948 10.1016/j.joca.2015.01.012 25655679 PMC4459965

[24] Zhu J Zhu Y Xiao W Hu Y Li Y Instability and excessive mechanical loading mediate subchondral bone changes to induce osteoarthritis Ann Transl Med 2020 8 6 350 10.21037/atm.2020.02.103 32355794 PMC7186756

[25] Kalu DN The ovariectomized rat model of postmenopausal bone loss Bone Miner 1991 15 3 175 191 10.1016/0169-6009(91)90124-i 1773131

[26] Yousefzadeh N Kashfi K Jeddi S Ghasemi A Ovariectomized rat model of osteoporosis: a practical guide EXCLI J 2020 19 89 107 10.17179/excli2019-1990 32038119 PMC7003643

[27] DiFrancesco L Sokoloff L Lipochondral degeneration of capsular tissue in osteoarthritic hips Am J Surg Pathol 1995 19 3 278 283 10.1097/00000478-199503000-00005 7872426

[28] Campbell TM Trudel G Laneuville O Knee flexion contractures in patients with osteoarthritis: clinical features and histologic characterization of the posterior capsule PM R 2015 7 5 466 473 10.1016/j.pmrj.2014.12.001 25511691

[29] Limberg AK Salib CG Tibbo ME et al. Immune cell populations differ in patients undergoing revision total knee arthroplasty for arthrofibrosis Sci Rep 2022 12 1 22627 10.1038/s41598-022-22175-3 36587032 PMC9805429

[30] Voelker A Schroeter F Steinke H Heyde CE Degeneration of the lumbar spine and its relation to the expression of collagen and elastin in facet joint capsules and ligament flavum Acta Orthop Traumatol Turc 2022 56 3 210 216 10.5152/j.aott.2022.21314 35703510 PMC9612638

[31] Herbert C Jayson MI Bailey AJ Joint capsule collagen in osteoarthrosis Ann Rheum Dis 1973 32 6 510 514 10.1136/ard.32.6.510 4760475 PMC1006161

[32] Cameron HU Macnab I Scanning electron microscopic studies of the hip joint capsule and synovial membrane Can J Surg 1973 16 388 392 4748419

[33] Heinegård D Hernborg J Lundberg BJ The glycosaminoglycans of the human joint capsule: isolation and characterizaion Arthritis Rheum 1968 11 6 787 795 10.1002/art.1780110608 4236075

[34] Belluzzi E Macchi V Fontanella CG et al. Infrapatellar fat pad gene expression and protein production in patients with and without osteoarthritis Int J Mol Sci 2020 21 17 6016 10.3390/ijms21176016 32825633 PMC7503946

[35] Grevenstein D Heilig J Dargel J et al. COMP in the infrapatellar fat pad-results of a prospective histological, immunohistological, and biochemical case-control study J Orthop Res 2020 38 4 747 758 10.1002/jor.24514 31696983

[36] Cheng XG Brys P Nijs J et al. Radiological prevalence of lumbar intervertebral disc calcification in the elderly: an autopsy study Skel Radiol 1996 25 3 231 235 10.1007/s002560050070 8741057

[37] Kumagai K Sakai K Kusayama Y et al. The extent of degeneration of cruciate ligament is associated with chondrogenic differentiation in patients with osteoarthritis of the knee Osteoarthr Cartil 2012 20 11 1258 1267 10.1016/j.joca.2012.07.013 22846713

[38] Komro J Gonzales J Marberry K Main DC Cramberg M Kondrashov P Fibrocartilaginous metaplasia and neovascularization of the anterior cruciate ligament in patients with osteoarthritis Clin Anat 2020 33 6 899 905 10.1002/ca.23590 32243680

[39] Nakamura Y Ogawa H Sohmiya K et al. Relationship between histological changes of the anterior cruciate ligament and knee function in osteoarthritis patients Orthop Traumatol Surg Res 2022 108 8 103341 10.1016/j.otsr.2022.103341 35643361

[40] Akisue T Stulberg BN Bauer TW McMahon JT Wilde AH Kurosaka M Histologic evaluation of posterior cruciate ligaments from osteoarthritic knees Clin Orthop Relat Res 2002 400 165 173 10.1097/00003086-200207000-00021 12072759

[41] Zhu J Zhang X Ma Y Zhou C Ao Y Ultrastructural and morphological characteristics of human anterior cruciate ligament and hamstring tendons Anat Rec (Hoboken) 2012 295 9 1430 1436 10.1002/ar.22527 22807249

[42] Levy YD Hasegawa A Patil S Koziol JA Lotz MK D’Lima DD Histopathological changes in the human posterior cruciate ligament during aging and osteoarthritis: correlations with anterior cruciate ligament and cartilage changes Ann Rheum Dis 2013 72 2 271 277 10.1136/annrheumdis-2012-201730 22872023 PMC3538921

[43] Marczak D Kowalczewski J Okoń T Synder M Sibiński M An evaluation of the posterior cruciate ligament function in total knee arthroplasty with regard to its morphology and clinical properties Folia Morphol (Warsz) 2017 76 1 94 99 10.5603/FM.a2016.0047 27665954

[44] Nakahara H Hasegawa A Otabe K et al. Transcription factor Mohawk and the pathogenesis of human anterior cruciate ligament degradation Arthritis Rheum 2013 65 8 2081 2089 10.1002/art.38020 23686683 PMC3840305

[45] Abdul Sahib NS Al-Sharqi SAH Wahab MS Study histopathological changes in the anterior and posterior cruciate ligament after knee replacement: correlations with vitamin D, calcium and c-reactive protein in iraqi patients with osteoarthritis Pak J Biotechnol 2017 14 393 400

[46] Allain J Goutallier D Voisin MC Macroscopic and histological assessments of the cruciate ligaments in arthrosis of the knee Acta Orthop Scand 2001 72 3 266 269 10.1080/00016470152846592 11480602

[47] Martins GC Camanho G Rodrigues MI Filho LFM Demange MK Histopathological analysis of the posterior cruciate ligament in primary osteoarthritis Eur J Orthop Surg Traumatol 2018 28 4 691 699 10.1007/s00590-018-2136-8 29417349

[48] Nelissen RG Hogendoorn PC Retain or sacrifice the posterior cruciate ligament in total knee arthroplasty? A histopathological study of the cruciate ligament in osteoarthritic and rheumatoid disease J Clin Pathol 2001 54 5 381 384 10.1136/jcp.54.5.381 11328838 PMC1731430

[49] Rajgopal A Vasdev N Pathak A Gautam D Vasdev A Histological changes and neural elements in the posterior cruciate ligament in osteoarthritic knees J Orthop Surg (Hong Kong) 2014 22 2 142 145 10.1177/230949901402200204 25163942

[50] Tokumoto M Nakasa T Shirakawa Y et al. The role of substance P on maintaining ligament homeostasis by inhibiting endochondral ossification during osteoarthritis progression Connect Tissue Res 2023 64 1 82 92 10.1080/03008207.2022.2099847 35856812

[51] Doerschuk SH Hicks DG Chinchilli VM Pellegrini VD Histopathology of the palmar beak ligament in trapeziometacarpal osteoarthritis J Hand Surg Am 1999 24 3 496 504 10.1053/jhsu.1999.0496 10357527

[52] Mobargha N Ludwig C Ladd AL Hagert E Ultrastructure and innervation of thumb carpometacarpal ligaments in surgical patients with osteoarthritis Clin Orthop Relat Res 2014 472 4 1146 1154 10.1007/s11999-013-3083-7 23761171 PMC3940730

[53] Suga Y Shigematsu H Tanaka M et al. Factors associated with the increased risk of atlantoaxial osteoarthritis: a retrospective study Eur Spine J 2022 31 12 3418 3425 10.1007/s00586-022-07414-5 36260133

[54] Sun Y Mauerhan DR Kneisl JS et al. Histological examination of collagen and proteoglycan changes in osteoarthritic menisci Open Rheumatol J 2012 6 1 24 32 10.2174/1874312901206010024 22550551 PMC3339434

[55] Kodama Y Furumatsu T Maehara A Ozaki T Composition of cell clusters in torn menisci and their extracellular matrix components Acta Med Okayama 2018 72 5 499 506 10.18926/AMO/56248 30369607

[56] Numpaisal PO Jiang CC Hsieh CH Chiang H Chien CL Prospective application of partially digested autologous chondrocyte for meniscus tissue engineering Pharmaceutics 2022 14 3 605 10.3390/pharmaceutics14030605 35335980 PMC8952194

[57] Melrose J Fuller ES Roughley PJ et al. Fragmentation of decorin, biglycan, lumican and keratocan is elevated in degenerate human meniscus, knee and hip articular cartilages compared with age-matched macroscopically normal and control tissues Arthritis Res Ther 2008 10 4 R79 10.1186/ar2453 18620607 PMC2575625

[58] Battistelli M Favero M Burini D et al. Morphological and ultrastructural analysis of normal, injured and osteoarthritic human knee menisci Eur J Histochem 2019 63 1 11 10.4081/ejh.2019.2998 30739432 PMC6379780

[59] McDaniel D Tilton E Dominick K et al. Histological characteristics of knee menisci in patients with osteoarthritis Clin Anat 2017 30 6 805 810 10.1002/ca.22920 28524271

[60] Hellberg I Karjalainen V-P Finnilä MAJ et al. 3D analysis and grading of calcifications from ex vivo human meniscus Osteoarthritis Cartilage 2023 31 4 482 492 10.1016/j.joca.2022.10.016 36356928 PMC7614369

[61] Abraham AC Pauly HM Donahue TLH Deleterious effects of osteoarthritis on the structure and function of the meniscal enthesis Osteoarthritis Cartilage 2014 22 2 275 283 10.1016/j.joca.2013.11.013 24316288 PMC3923977

[62] Dessombz A Nguyen C Ea H-K et al. Combining μX-ray fluorescence, μXANES and μXRD to shed light on Zn2+ cations in cartilage and meniscus calcifications J Trace Elem Med Biol 2013 27 4 326 333 10.1016/j.jtemb.2013.02.001 23582484

[63] Johnson K Hashimoto S Lotz M Pritzker K Goding J Terkeltaub R Up-regulated expression of the phosphodiesterase nucleotide pyrophosphatase family member PC-1 is a marker and pathogenic factor for knee meniscal cartilage matrix calcification Arthritis Rheum 2001 44 5 1071 1081 10.1002/1529-0131(200105)44:5<1071::AID-ANR187>3.0.CO;2-3 11352238

[64] Kiraly AJ Roberts A Cox M Mauerhan D Hanley E Sun Y Comparison of meniscal cell-mediated and chondrocyte-mediated calcification Open Orthop J 2017 11 225 233 10.2174/1874325001711010225 28567149 PMC5420175

[65] López-Franco M López-Franco O Murciano-Antón MA et al. Meniscal degeneration in human knee osteoarthritis: in situ hybridization and immunohistochemistry study Arch Orthop Trauma Surg 2016 136 2 175 183 10.1007/s00402-015-2378-4 26667622

[66] Park DY Min B-H Choi BH et al. The degeneration of meniscus roots is accompanied by fibrocartilage formation, which may precede meniscus root tears in osteoarthritic knees Am J Sports Med 2015 43 12 3034 3044 10.1177/0363546515605086 26430056

[67] Sun Y Mauerhan DR Honeycutt PR et al. Calcium deposition in osteoarthritic meniscus and meniscal cell culture Arthritis Res Ther 2010 12 2 R56 10.1186/ar2968 20353559 PMC2888206

[68] Takahashi M Suzuki M Kushida K Hoshino H Inoue T The effect of aging and osteoarthritis on the mature and senescent cross-links of collagen in human meniscus Arthroscopy 1998 14 4 366 372 10.1016/s0749-8063(98)70003-9 9620647

[69] Zhang D Cheriyan T Martin SD Schmid TM Spector M Lubricin distribution in the menisci and labra of human osteoarthritic joints Cartilage 2012 3 2 165 172 10.1177/1947603511429699 26069629 PMC4297123

[70] Prokopi N Andrikopoulos KS Beobide AS Voyiatzis GA Papachristou DJ Collagen orientation probed by polarized Raman spectra can serve as differential diagnosis indicator between different grades of meniscus degeneration Sci Rep 2021 11 1 20299 10.1038/s41598-021-99569-2 34645874 PMC8514572

[71] Sirotti S Becce F Sconfienza LM et al. Reliability and diagnostic accuracy of radiography for the diagnosis of calcium pyrophosphate deposition: performance of the novel definitions developed by an international multidisciplinary working group Arthritis Rheumatol 2023 75 4 630 638 10.1002/art.42368 36122187

[72] Roller BL Monibi FA Stoker AM Kuroki K Bal BS Cook JL Characterization of knee meniscal pathology: correlation of gross, histologic, biochemical, molecular, and radiographic measures of disease J Knee Surg 2015 28 2 175 182 10.1055/s-0034-1376333 24807193

[73] Ghosh P Ingman AM Taylor TK Variations in collagen, non-collagenous proteins, and hexosamine in menisci derived from osteoarthritic and rheumatoid arthritic knee joints J Rheumatol 1975 2 1 100 107 1185729

[74] Son M Goodman SB Chen W Hargreaves BA Gold GE Levenston ME Regional variation in T1ρ and T2 times in osteoarthritic human menisci: correlation with mechanical properties and matrix composition Osteoarthritis Cartilage 2013 21 6 796 805 10.1016/j.joca.2013.03.002 23499673 PMC3909565

[75] Warnecke D Balko J Haas J et al. Degeneration alters the biomechanical properties and structural composition of lateral human menisci Osteoarthritis Cartilage 2020 28 11 1482 1491 10.1016/j.joca.2020.07.004 32739340

[76] Mine T Ihara K Kawamura H Date R Umehara K Collagen expression in various degenerative meniscal changes: an immunohistological study J Orthop Surg (Hong Kong) 2013 21 2 216 220 10.1177/230949901302100221 24014788

[77] Sladojević I Krivokuća Z Gajanin V Manojlović S Expression of collagen type I in unaltered and osteoarthritic menisci of knee joint Med Pregl 2016 69 1–2 16 23 10.2298/mpns1602016s 27498529

[78] Hino T Furumatsu T Miyazawa S et al. A histological study of the medial meniscus posterior root tibial insertion Connect Tissue Res 2020 61 6 546 553 10.1080/03008207.2019.1631298 31181971

[79] Ishizuka S Sakai T Hiraiwa H et al. Hypoxia-inducible factor-2α induces expression of type X collagen and matrix metalloproteinases 13 in osteoarthritic meniscal cells Inflamm Res 2016 65 6 439 448 10.1007/s00011-016-0926-1 26892680

[80] Katsuragawa Y Saitoh K Tanaka N et al. Changes of human menisci in osteoarthritic knee joints Osteoarthritis Cartilage 2010 18 9 1133 1143 10.1016/j.joca.2010.05.017 20633672

[81] Jacquet C Erivan R Argenson JN Parratte S Ollivier M Effect of 3 preservation methods (freezing, cryopreservation, and freezing + irradiation) on human menisci ultrastructure: an ex vivo comparative study with fresh tissue as a gold standard Am J Sports Med 2018 46 12 2899 2904 10.1177/0363546518790504 30141963

[82] Karjalainen V-P Kestilä I Finnilä MA et al. Quantitative three-dimensional collagen orientation analysis of human meniscus posterior horn in health and osteoarthritis using micro-computed tomography Osteoarthritis Cartilage 2021 29 5 762 772 10.1016/j.joca.2021.01.009 33588085 PMC7610734

[83] Atik OŞ Erdoğan D Seymen CM Bozkurt HH Kaplanoğlu GT Is there crosstalk between subchondral bone, cartilage, and meniscus in the pathogenesis of osteoarthritis? Eklem Hast Cerr 2016 27 2 62 67 10.5606/ehc.2016.14 27499316

[84] Haut Donahue TL Pauly HM Osteoarthritic meniscal entheses exhibit altered collagen fiber orientation Connect Tissue Res 2022 63 2 151 155 10.1080/03008207.2021.1890723 33588665

[85] Nagata N Koshino T Saito T Up-regulation of CD44-positive cells in medial meniscus of medial compartmental osteoarthritis of the knee Knee 2000 7 1 3 9 10.1016/S0968-0160(99)00029-0

[86] Wang J Roberts S Kuiper JH et al. Characterization of regional meniscal cell and chondrocyte phenotypes and chondrogenic differentiation with histological analysis in osteoarthritic donor-matched tissues Sci Rep 2020 10 1 21658 10.1038/s41598-020-78757-6 33303888 PMC7730426

[87] Gouldin AG Patel NK Golladay GJ Puetzer JL Advanced glycation end-product accumulation differs by location and sex in aged osteoarthritic human menisci Osteoarthritis Cartilage 2023 31 3 363 373 10.1016/j.joca.2022.11.012 36494052 PMC10088070

[88] Fischenich KM Lewis J Kindsfater KA Bailey TS Haut Donahue TL Effects of degeneration on the compressive and tensile properties of human meniscus J Biomech 2015 48 8 1407 1411 10.1016/j.jbiomech.2015.02.042 25770751

[89] Kwok J Grogan S Meckes B Arce F Lal R D’Lima D Atomic force microscopy reveals age-dependent changes in nanomechanical properties of the extracellular matrix of native human menisci: implications for joint degeneration and osteoarthritis Nanomedicine 2014 10 8 1777 1785 10.1016/j.nano.2014.06.010 24972006 PMC4374607

[90] Pordzik J Bernstein A Mayr HO et al. Analysis of proteoglycan content and biomechanical properties in arthritic and arthritis-free menisci Appl Sci (Basel) 2020 10 24 9012 10.3390/app10249012

[91] Fuhrmann IK Steinhagen J Rüther W Schumacher U Comparative immunohistochemical evaluation of the zonal distribution of extracellular matrix and inflammation markers in human meniscus in osteoarthritis and rheumatoid arthritis Acta Histochem 2015 117 3 243 254 10.1016/j.acthis.2014.12.009 25827912

[92] Monibi FA Pannellini T Otero M Warren RF Rodeo SA Histologic and molecular features in pathologic human menisci from knees with and without osteoarthritis J Orthop Res 2022 40 2 504 512 10.1002/jor.25047 33792974 PMC8484374

[93] Karube S Shoji H Compositional changes of glycosaminoglycans of the human menisci with age and degenerative joint disease Nippon Seikeigeka Gakkai Zasshi 1982 56 1 51 57 6896062

[94] Masuda I Ishikawa K Usuku G A histologic and immunohistochemical study of calcium pyrophosphate dihydrate crystal deposition disease Clin Orthop Relat Res 1991 263 272 287 1993383

[95] Musumeci G Trovato FM Loreto C et al. Lubricin expression in human osteoarthritic knee meniscus and synovial fluid: a morphological, immunohistochemical and biochemical study Acta Histochem 2014 116 5 965 972 10.1016/j.acthis.2014.03.011 24932985

[96] Jacquet C Erivan R Sharma A et al. Preservation methods influence the biomechanical properties of human lateral menisci: an ex vivo comparative study of 3 methods Orthop J Sports Med 2019 7 4 2325967119841622 10.1177/2325967119841622 31065555 PMC6488788

[97] Folkesson E Turkiewicz A Ali N et al. Proteomic comparison of osteoarthritic and reference human menisci using data-independent acquisition mass spectrometry Osteoarthritis Cartilage 2020 28 8 1092 1101 10.1016/j.joca.2020.05.001 32407894 PMC7397514

[98] Roller BL Monibi F Stoker AM Bal BS Stannard JP Cook JL Characterization of meniscal pathology using molecular and proteomic analyses J Knee Surg 2015 28 6 496 505 10.1055/s-0034-1394164 25340674

[99] Park J Lee H-S Go E-B et al. Proteomic analysis of the meniscus cartilage in osteoarthritis Int J Mol Sci 2021 22 15 8181 10.3390/ijms22158181 34360947 PMC8348647

[100] Fink B Egl M Singer J Fuerst M Bubenheim M Neuen-Jacob E Morphologic changes in the vastus medialis muscle in patients with osteoarthritis of the knee Arthritis Rheum 2007 56 11 3626 3633 10.1002/art.22960 17968889

[101] Noehren B Kosmac K Walton RG et al. Alterations in quadriceps muscle cellular and molecular properties in adults with moderate knee osteoarthritis Osteoarthritis Cartilage 2018 26 10 1359 1368 10.1016/j.joca.2018.05.011 29800621 PMC7050996

[102] Serrão PR Vasilceac FA Gramani-Say K et al. Expression of receptors of advanced glycation end product (RAGE) and types I, III and IV collagen in the vastus lateralis muscle of men in early stages of knee osteoarthritis Connect Tissue Res 2014 55 5–6 331 338 10.3109/03008207.2014.947368 25039336

[103] Mattiello-Sverzut AC Petersen SG Kjaer M Mackey AL Morphological adaptation of muscle collagen and receptor of advanced glycation end product (RAGE) in osteoarthritis patients with 12 weeks of resistance training: influence of anti-inflammatory or glucosamine treatment Rheumatol Int 2013 33 9 2215 2224 10.1007/s00296-013-2698-z 23443332

[104] Krawetz RJ Wu YE Bertram KL et al. Synovial mesenchymal progenitor derived aggrecan regulates cartilage homeostasis and endogenous repair capacity Cell Death Dis 2022 13 5 470 10.1038/s41419-022-04919-1 35585042 PMC9117284

[105] Rafael MS Cavaco S Viegas CSB et al. Insights into the association of Gla‐rich protein and osteoarthritis, novel splice variants and γ‐carboxylation status Mol Nutr Food Res 2014 58 8 1636 1646 10.1002/mnfr.201300941 24867294

[106] Ea H-K Chobaz V Nguyen C et al. Pathogenic role of basic calcium phosphate crystals in destructive arthropathies PLoS One 2013 8 2 e57352 10.1371/journal.pone.0057352 23468973 PMC3585350

[107] Nakashima K Koshino T Saito T Synovial immunohistochemical changes after high tibial osteotomy for osteoarthritis of the knee. Two-year prospective follow-up Bull Hosp Jt Dis 1998 57 4 187 194 9926257

[108] Saito I Koshino T Nakashima K Uesugi M Saito T Increased cellular infiltrate in inflammatory synovia of osteoarthritic knees Osteoarthritis Cartilage 2002 10 2 156 162 10.1053/joca.2001.0494 11869075

[109] Richardot P Charni-Ben Tabassi N Toh L et al. Nitrated type III collagen as a biological marker of nitric oxide-mediated synovial tissue metabolism in osteoarthritis Osteoarthritis Cartilage 2009 17 10 1362 1367 10.1016/j.joca.2009.04.024 19467351

[110] Ene R Sinescu RD Ene P Cîrstoiu MM Cîrstoiu FC Synovial inflammation in patients with different stages of knee osteoarthritis Rom J Morphol Embryol 2015 56 1 169 173 25826502

[111] Kaufmann J Mueller A Voigt A et al. Hydroxypyridinium collagen crosslinks in serum, urine, synovial fluid and synovial tissue in patients with rheumatoid arthritis compared with osteoarthritis Rheumatol (Oxford) 2003 42 2 314 320 10.1093/rheumatology/keg102 12595629

[112] Takahashi M Kushida K Hoshino H et al. Concentrations of pyridinoline and deoxypyridinoline in joint tissues from patients with osteoarthritis or rheumatoid arthritis Ann Rheum Dis 1996 55 5 324 327 10.1136/ard.55.5.324 8660108 PMC1010171

[113] Di Cesare PE Fang C Leslie MP et al. Localization and expression of cartilage oligomeric matrix protein by human rheumatoid and osteoarthritic synovium and cartilage J Orthop Res 1999 17 3 437 445 10.1002/jor.1100170321 10376735

[114] Cillero-Pastor B Eijkel GB Blanco FJ Heeren RMA Protein classification and distribution in osteoarthritic human synovial tissue by matrix-assisted laser desorption ionization mass spectrometry imaging Anal Bioanal Chem 2015 407 8 2213 2222 10.1007/s00216-014-8342-2 25504090

[115] Cutolo M Picasso M Ponassi M Sun MZ Balza E Tenascin and fibronectin distribution in human normal and pathological synovium J Rheumatol 1992 19 9 1439 1447 1279171

[116] Fan L Wang Q Liu R et al. Citrullinated fibronectin inhibits apoptosis and promotes the secretion of pro-inflammatory cytokines in fibroblast-like synoviocytes in rheumatoid arthritis Arthritis Res Ther 2012 14 6 R266 10.1186/ar4112 23217276 PMC3674622

[117] Kragstrup TW Sohn DH Lepus CM et al. Fibroblast-like synovial cell production of extra domain A fibronectin associates with inflammation in osteoarthritis BMC Rheumatol 2019 3 46 10.1186/s41927-019-0093-4 31819923 PMC6886182

[118] Nikkari L Haapasalmi K Aho H et al. Localization of the alpha V subfamily of integrins and their putative ligands in synovial lining cell layer J Rheumatol 1995 22 1 16 23 7535359

[119] Nishida K Inoue H Toda K Murakami T Localization of the glycosaminoglycans in the synovial tissues from osteoarthritic knees Acta Med Okayama 1995 49 6 287 294 10.18926/AMO/30384 8770237

[120] Wang X Dong C Li N et al. Modulation of TGF‑β activity by latent TGF‑β‑binding protein 1 in human osteoarthritis fibroblast‑like synoviocytes Mol Med Rep 2018 17 1893 1900 10.3892/mmr.2017.8086 29257223

[121] Turdean SG Jung I Gurzu S et al. Histopathological evaluation and expression of the pluripotent mesenchymal stem cell-like markers CD105 and CD44 in the synovial membrane of patients with primary versus secondary hip osteoarthritis J Investig Med 2017 65 2 363 369 10.1136/jim-2016-000244 27803113

[122] Konttinen YT Li TF Mandelin J et al. Hyaluronan synthases, hyaluronan, and its CD44 receptor in tissue around loosened total hip prostheses J Pathol 2001 194 3 384 390 10.1002/1096-9896(200107)194:3<384::AID-PATH896>3.0.CO;2-8 11439372

[123] Christensen AF Sorensen GL Junker K et al. Site-specific absence of microfibrillar-associated protein 4 (MFAP4) from the internal elastic membrane of arterioles in the rheumatoid arthritis synovial membrane: an immunohistochemical study in patients with advanced rheumatoid arthritis versus osteoarthritis APMIS 2019 127 8 588 593 10.1111/apm.12974 31233243

[124] Li TF Xu JW Santavirta S et al. Distribution of fibronectins and their integrin receptors in interface tissue from aseptic loosening of hip prostheses Clin Exp Rheumatol 2000 18 2 221 225 10812495

[125] Konttinen YT Li TF Xu JW et al. Expression of laminins and their integrin receptors in different conditions of synovial membrane and synovial membrane-like interface tissue Ann Rheum Dis 1999 58 11 683 690 10.1136/ard.58.11.683 10531072 PMC1752798

[126] van Linthoudt D Beutler A Clayburne G Sieck M Fernandes L Schumacher HR Morphometric studies on synovium in advanced osteoarthritis: is there an association between apatite-like material and collagen deposits? Clin Exp Rheumatol 1997 15 5 493 497 9307856

[127] Pollock LE Lalor P Revell PA Type IV collagen and laminin in the synovial intimal layer: an immunohistochemical study Rheumatol Int 1990 9 6 277 280 10.1007/BF00541324 2315607

[128] Mapp PI Revell PA Fibronectin production by synovial intimal cells Rheumatol Int 1985 5 5 229 237 10.1007/BF00541341 4070925

[129] Scott DL Wainwright AC Walton KW Williamson N Significance of fibronectin in rheumatoid arthritis and osteoarthrosis Ann Rheum Dis 1981 40 2 142 153 10.1136/ard.40.2.142 7013719 PMC1000696

[130] Worrall JG Wilkinson LS Bayliss MT Edwards JCW Zonal distribution of chondroitin-4-sulphate/dermatan sulphate and chondroitin-6-sulphate in normal and diseased human synovium Ann Rheum Dis 1994 53 1 35 38 10.1136/ard.53.1.35 8311553 PMC1005240

[131] Rinaldi N Barth TF Weis D et al. Loss of laminin and of the laminin receptor integrin subunit alpha 6 in situ correlates with cytokine induced down regulation of alpha 6 on fibroblast-like synoviocytes from rheumatoid arthritis Ann Rheum Dis 1998 57 9 559 565 10.1136/ard.57.9.559 9849316 PMC1752734

[132] Dijkgraaf LC Liem RSB de Bont LGM Ultrastructural characteristics of the synovial membrane in osteoarthritic temporomandibular joints J Oral Maxillofac Surg 1997 55 11 1269 1279 10.1016/S0278-2391(97)90183-X 9371119

[133] Okamoto K Kiga N Shinohara Y Tojyo I Fujita S Effect of interleukin-1beta and dehydroepiandrosterone on the expression of lumican and fibromodulin in fibroblast-like synovial cells of the human temporomandibular joint Eur J Histochem 2015 59 1 2440 10.4081/ejh.2015.2440 25820556 PMC4378210

[134] Schneider M Voss B Rauterberg J et al. Basement membrane proteins in synovial membrane: distribution in rheumatoid arthritis and synthesis by fibroblast-like cells Clin Rheumatol 1994 13 1 90 97 10.1007/BF02229873 8187452

[135] Klareskog L Johnell O Hulth A Holmdahl R Rubin K Reactivity of monoclonal anti‐type II collagen antibodies with cartilage and synovial tissue in rheumatoid arthritis and osteoarthritis Arthritis Rheum 1986 29 6 730 738 10.1002/art.1780290605 2424461

[136] Scott DL Salmon M Morris CJ Wainwright AC Walton KW Laminin and vascular proliferation in rheumatoid arthritis Ann Rheum Dis 1984 43 4 551 555 10.1136/ard.43.4.551 6383233 PMC1001406

[137] Chang X Yamada R Suzuki A Kochi Y Sawada T Yamamoto K Citrullination of fibronectin in rheumatoid arthritis synovial tissue Rheumatology (Oxford) 2005 44 11 1374 1382 10.1093/rheumatology/kei023 16105911

[138] Hino K Shiozawa S Kuroki Y et al. EDA-containing fibronectin is synthesized from rheumatoid synovial fibroblast-like cells Arthritis Rheum 1995 38 5 678 683 10.1002/art.1780380516 7748223

[139] Kriegsmann J Berndt A Hansen T et al. Expression of fibronectin splice variants and oncofetal glycosylated fibronectin in the synovial membranes of patients with rheumatoid arthritis and osteoarthritis Rheumatol Int 2004 24 1 25 33 10.1007/s00296-003-0316-1 12712258

[140] Itokazu M Shinozaki M Ohno T Quantitative analysis of hyaluronan in the synovial tissues of patients with joint disorders Clin Rheumatol 1998 17 3 261 262 10.1007/BF01451063 9694068

[141] Santiago B Baleux F Palao G et al. CXCL12 is displayed by rheumatoid endothelial cells through its basic amino-terminal motif on heparan sulfate proteoglycans Arthritis Res Ther 2006 8 2 R43 10.1186/ar1900 16507142 PMC1526602

[142] Poduval P Sillat T Virtanen I Dabagh M Konttinen YT Immigration check for neutrophils in RA lining: laminin alpha5 low expression regions act as exit points Scand J Rheumatol 2010 39 2 132 140 10.3109/03009740903198980 20059371

[143] Ren X Geng M Xu K et al. Quantitative proteomic analysis of synovial tissue reveals that upregulated OLFM4 aggravates inflammation in rheumatoid arthritis J Proteome Res 2021 20 10 4746 4757 10.1021/acs.jproteome.1c00399 34496567

[144] Worrall JG Bayliss MT Edwards JCW Morphological localization of hyaluronan in normal and diseased synovium J Rheumatol 1991 18 10 1466 1472 1765969

[145] Meknas K Johansen O Steigen SE Olsen R Jørgensen L Kartus J Could tendinosis be involved in osteoarthritis? Scand J Med Sci Sports 2012 22 5 627 634 10.1111/j.1600-0838.2010.01287.x 21410541

[146] Ibrahim M Kartus JT Steigen SE Olsen R Meknas K More tendon degeneration in patients with shoulder osteoarthritis Knee Surg Sports Traumatol Arthrosc 2019 27 1 267 275 10.1007/s00167-018-5186-x 30284007

[147] Expósito Molinero MR de Miguel Mendieta E Discriminant validity study of Achilles enthesis ultrasound Reum Clin 2016 12 4 206 209 10.1016/j.reuma.2015.08.006 26573883

[148] Mazzocca AD McCarthy MBR Ledgard FA et al. Histomorphologic changes of the long head of the biceps tendon in common shoulder pathologies Arthroscopy 2013 29 6 972 981 10.1016/j.arthro.2013.02.002 23571131

[149] Ibrahim M Hedlundh U Sernert N et al. Histological and ultrastructural degenerative findings in the gluteus medius tendon after hip arthroplasty J Orthop Surg Res 2021 16 1 339 10.1186/s13018-021-02434-1 34039378 PMC8152320

[150] Loeser RF Olex AL McNulty MA et al. Disease progression and phasic changes in gene expression in a mouse model of osteoarthritis PLoS One 2013 8 1 e54633 10.1371/journal.pone.0054633 23382930 PMC3557277

[151] Ramos-Mucci L Javaheri B van’t Hof R et al. Meniscal and ligament modifications in spontaneous and post-traumatic mouse models of osteoarthritis Arthritis Res Ther 2020 22 1 171 10.1186/s13075-020-02261-5 32678020 PMC7364489

[152] Walton M Degenerative joint disease in the mouse knee; radiological and morphological observations J Pathol 1977 123 2 97 107 10.1002/path.1711230206 592018

[153] Anderson-MacKenzie JM Billingham ME Bailey AJ Collagen remodeling in the anterior cruciate ligament associated with developing spontaneous murine osteoarthritis Biochem Biophys Res Commun 1999 258 3 763 767 10.1006/bbrc.1999.0713 10329460

[154] Cui P Sun B-H Dai Y-F et al. Healing of the torn anterior horn of rabbit medial meniscus to bone after transtibial pull-out repair and autologous platelet-rich plasma gel injection Orthop Surg 2023 15 2 617 627 10.1111/os.13622 36573287 PMC9891914

[155] Miller D DeSutter C Scott A et al. Vascular structure and function in the medial collateral ligament of anterior cruciate ligament transected rabbit knees J Orthop Res 2014 32 9 1104 1110 10.1002/jor.22643 24909758

[156] Funakoshi Y Hariu M Tapper JE et al. Periarticular ligament changes following ACL/MCL transection in an ovine stifle joint model of osteoarthritis J Orthop Res 2007 25 8 997 1006 10.1002/jor.20370 17436314

[157] Barton KI Heard BJ Kroker A et al. Structural consequences of a partial anterior cruciate ligament injury on remaining joint integrity: evidence for ligament and bone changes over time in an ovine model Am J Sports Med 2021 49 3 637 648 10.1177/0363546520985279 33523721

[158] Bedingfield SK Colazo JM Di Francesco M et al. Top-down fabricated microPlates for prolonged, intra-articular matrix metalloproteinase 13 siRNA nanocarrier delivery to reduce post-traumatic osteoarthritis ACS Nano 2021 15 9 14475 14491 10.1021/acsnano.1c04005 34409835 PMC9074946

[159] Muschter D Fleischhauer L Taheri S Schilling AF Clausen-Schaumann H Grässel S Sensory neuropeptides are required for bone and cartilage homeostasis in a murine destabilization-induced osteoarthritis model Bone 2020 133 115181 10.1016/j.bone.2019.115181 31926346

[160] Catheline SE Bell RD Oluoch LS et al. IKKβ-NF-κB signaling in adult chondrocytes promotes the onset of age-related osteoarthritis in mice Sci Signal 2021 14 701 eabf3535 10.1126/scisignal.abf3535 34546791 PMC8734558

[161] Lee KI Gamini R Olmer M et al. Mohawk is a transcription factor that promotes meniscus cell phenotype and tissue repair and reduces osteoarthritis severity Sci Transl Med 2020 12 567 28 10.1126/scitranslmed.aan7967 33115953 PMC7955769

[162] Le Graverand MPH Sciore P Eggerer J Formation and phenotype of cell clusters in osteoarthritic meniscus Arthritis Rheum 2001 44 8 1808 1818 10.1002/1529-0131(200108)44:8<1808::AID-ART318>3.0.CO;2-B 11508433

[163] Hellio Le Graverand MP Vignon E Otterness IG Hart DA Early changes in lapine menisci during osteoarthritis development: Part I: Cellular and matrix alterations Osteoarthritis Cartilage 2001 9 1 56 64 10.1053/joca.2000.0350 11178948

[164] Zhao J Huang S Zheng J et al. Changes of rabbit meniscus influenced by hyaline cartilage injury of osteoarthritis Int J Clin Exp Med 2014 7 9 2948 2956 25356168 PMC4211818

[165] Levillain A Magoariec H Boulocher C Decambron A Viateau V Hoc T Viscoelastic properties of rabbit osteoarthritic menisci: A correlation with matrix alterations J Mech Behav Biomed Mater 2017 65 1 10 10.1016/j.jmbbm.2016.08.015 27543842

[166] Levillain A Magoariec H Boulocher C Decambron A Viateau V Hoc T Effects of a viscosupplementation therapy on rabbit menisci in an anterior cruciate ligament transection model of osteoarthritis J Biomech 2017 58 147 154 10.1016/j.jbiomech.2017.04.034 28554494

[167] Endo J Sasho T Akagi R et al. Comparative analysis of gene expression between cartilage and menisci in early-phase osteoarthritis of the knee-an animal model study J Knee Surg 2018 31 7 664 669 10.1055/s-0037-1606549 28915521

[168] Bansal S Miller LM Patel JM et al. Transection of the medial meniscus anterior horn results in cartilage degeneration and meniscus remodeling in a large animal model J Orthop Res 2020 38 12 2696 2708 10.1002/jor.24694 32285971 PMC7735384

[169] Bansal S Meadows KD Miller LM et al. Six-month outcomes of clinically relevant meniscal injury in a large-animal model Orthop J Sports Med 2021 9 11 23259671211035444 10.1177/23259671211035444 34796238 PMC8593308

[170] Shi X Yu W Wang T et al. Electroacupuncture alleviates cartilage degradation: Improvement in cartilage biomechanics via pain relief and potentiation of muscle function in a rabbit model of knee osteoarthritis Biomed Pharmacother 2020 123 109724 10.1016/j.biopha.2019.109724 31918209

[171] Lee K Gang GG Kang YG Jung SS Park HG Jang JH Alleviation of osteoarthritis-induced pain and motor deficits in rats by a novel device for the intramuscular insertion of cog polydioxanone filament Appl Sci (Basel) 2021 11 22 10534 10.3390/app112210534

[172] Tavallaee G Lively S Rockel JS et al. Contribution of microRNA-27b-3p to synovial fibrotic responses in knee osteoarthritis Arthritis Rheumatol 2022 74 12 1928 1942 10.1002/art.42285 35791923 PMC10946865

[173] Gamal N Abou-Rabia NM El Ebiary FH Khalaf G Raafat MH The possible therapeutic role of platelet rich plasma on a model of osteoarthritis in male albino rat. Histological and immunohistochemical study Egypt J Histol 2019 42 3 554 566 10.21608/ejh.2019.9750.1090

[174] Zhang L Zhang L Huang Z et al. Increased HIF-1*α* in knee osteoarthritis aggravate synovial fibrosis via fibroblast-like synoviocyte pyroptosis Oxid Med Cell Longev 2019 2019 6326517 10.1155/2019/6326517 30755787 PMC6348923

[175] Zhang L Li X Zhang H et al. Agnuside alleviates synovitis and fibrosis in knee osteoarthritis through the inhibition of HIF-1*α* and NLRP3 inflammasome Mediators Inflamm 2021 2021 5534614 10.1155/2021/5534614 33814979 PMC7987448

[176] Li M Zhang L Liu Z et al. Sanse powder essential oil nanoemulsion negatively regulates TRPA1 by AMPK/mTOR signaling in synovitis: knee osteoarthritis rat model and fibroblast-like synoviocyte isolates Mediators Inflamm 2021 2021 4736670 10.1155/2021/4736670 34876884 PMC8645395

[177] Sriwatananukulkit O Desclaux S Tawonsawatruk T et al. Effectiveness of losartan on infrapatellar fat pad/synovial fibrosis and pain behavior in the monoiodoacetate-induced rat model of osteoarthritis pain Biomed Pharmacother 2023 158 114121 10.1016/j.biopha.2022.114121 36516695

[178] Zhang L Xing R Huang Z et al. Inhibition of synovial macrophage pyroptosis alleviates synovitis and fibrosis in knee osteoarthritis Mediators Inflamm 2019 2019 2165918 10.1155/2019/2165918 31582897 PMC6754937

[179] Zhang L Li M Li X et al. Characteristics of sensory innervation in synovium of rats within different knee osteoarthritis models and the correlation between synovial fibrosis and hyperalgesia J Adv Res 2022 35 141 151 10.1016/j.jare.2021.06.007 35003798 PMC8721247

[180] Li X Mei W Huang Z et al. Casticin suppresses monoiodoacetic acid-induced knee osteoarthritis through inhibiting HIF-1α/NLRP3 inflammasome signaling Int Immunopharmacol 2020 86 106745 10.1016/j.intimp.2020.106745 32622201

[181] Almasry SM Soliman HM El-Tarhouny SA Algaidi SA Ragab EM Platelet rich plasma enhances the immunohistochemical expression of platelet derived growth factor and vascular endothelial growth factor in the synovium of the meniscectomized rat models of osteoarthritis Ann Anat 2015 197 38 49 10.1016/j.aanat.2014.10.006 25466931

[182] Dai S Liang T Fujii T et al. Increased elastic modulus of the synovial membrane in a rat ACLT model of osteoarthritis revealed by atomic force microscopy Braz J Med Biol Res 2020 53 11 e10058 10.1590/1414-431X202010058 33053109 PMC7552902

[183] Bryk M Chwastek J Mlost J Kostrzewa M Starowicz K Sodium monoiodoacetate dose-dependent changes in matrix metalloproteinases and inflammatory components as prognostic factors for the progression of osteoarthritis Front Pharmacol 2021 12 643605 10.3389/fphar.2021.643605 33995052 PMC8113822

[184] Castrogiovanni P Di Rosa M Ravalli S et al. Moderate physical activity as a prevention method for knee osteoarthritis and the role of synoviocytes as biological key Int J Mol Sci 2019 20 3 511 10.3390/ijms20030511 30691048 PMC6387266

[185] Wei Q Kong N Liu X et al. Pirfenidone attenuates synovial fibrosis and postpones the progression of osteoarthritis by anti-fibrotic and anti-inflammatory properties in vivo and in vitro J Transl Med 2021 19 1 157 10.1186/s12967-021-02823-4 33874948 PMC8054406

[186] Lapadula G Nico B Cantatore FP La Canna R Roncali L Pipitone V Early ultrastructural changes of articular cartilage and synovial membrane in experimental vitamin A-induced osteoarthritis J Rheumatol 1995 22 10 1913 1921 8991991

[187] McErlain DD Appleton CTG Litchfield RB et al. Study of subchondral bone adaptations in a rodent surgical model of OA using in vivo micro-computed tomography Osteoarthritis Cartilage 2008 16 4 458 469 10.1016/j.joca.2007.08.006 17900933 PMC5130342

[188] Dabrowska S Ekiert-Radecka M Karbowniczek J et al. Calcification alters the viscoelastic properties of tendon fascicle bundles depending on matrix content Acta Biomater 2023 166 360 374 10.1016/j.actbio.2023.05.010 37172636

[189] Ricard-Blum S The collagen family Cold Spring Harb Perspect Biol 2011 3 1 a004978 10.1101/cshperspect.a004978 21421911 PMC3003457

[190] Chen D Smith LR Khandekar G et al. Distinct effects of different matrix proteoglycans on collagen fibrillogenesis and cell-mediated collagen reorganization Sci Rep 2020 10 1 19065 10.1038/s41598-020-76107-0 33149218 PMC7642422

[191] Naba A Ten years of extracellular matrix proteomics: accomplishments, challenges, and future perspectives Mol Cell Proteomics 2023 22 4 100528 10.1016/j.mcpro.2023.100528 36918099 PMC10152135

[192] Cope PJ Ourradi K Li Y Sharif M Models of osteoarthritis: the good, the bad and the promising Osteoarthritis Cartilage 2019 27 2 230 239 10.1016/j.joca.2018.09.016 30391394 PMC6350005

[193] Proffen BL McElfresh M Fleming BC Murray MM A comparative anatomical study of the human knee and six animal species Knee 2012 19 4 493 499 10.1016/j.knee.2011.07.005 21852139 PMC3236814

[194] Tschon M Contartese D Pagani S Borsari V Fini M Gender and sex are key determinants in osteoarthritis not only confounding variables. A systematic review of clinical data J Clin Med 2021 10 14 3178 10.3390/jcm10143178 34300344 PMC8303951

[195] Szilagyi IA Waarsing JH Schiphof D van Meurs JBJ Bierma-Zeinstra SMA Towards sex-specific osteoarthritis risk models: evaluation of risk factors for knee osteoarthritis in males and females Rheumatology (Oxford) 2022 61 2 648 657 10.1093/rheumatology/keab378 33895803 PMC8824415

[196] Karp NA Reavey N Sex bias in preclinical research and an exploration of how to change the status quo Br J Pharmacol 2019 176 21 4107 4118 10.1111/bph.14539 30418665 PMC6877896

[197] Pucha KA McKinney JM Fuller JM Willett NJ Characterization of OA development between sexes in the rat medial meniscal transection model Osteoarthr Cartil Open 2020 2 3 100066 10.1016/j.ocarto.2020.100066 36474679 PMC9718073

[198] Temp J Labuz D Negrete R Sunkara V Machelska H Pain and knee damage in male and female mice in the medial meniscal transection-induced osteoarthritis Osteoarthritis Cartilage 2020 28 4 475 485 10.1016/j.joca.2019.11.003 31830592

[199] Hwang HS Park IY Hong JI Kim JR Kim HA Comparison of joint degeneration and pain in male and female mice in DMM model of osteoarthritis Osteoarthritis Cartilage 2021 29 5 728 738 10.1016/j.joca.2021.02.007 33609695

[200] Malfait AM Miller RE Why we should study osteoarthritis pain in experimental models in both sexes Osteoarthritis Cartilage 2020 28 4 397 399 10.1016/j.joca.2019.12.008 31926266 PMC7108964

[201] Franke M Mancino C Taraballi F Reasons for the sex bias in osteoarthritis research: a review of preclinical studies Int J Mol Sci 2023 24 12 10386 10.3390/ijms241210386 37373536 PMC10299001

[202] Derfus BA Kurian JB Butler JJ et al. The high prevalence of pathologic calcium crystals in pre-operative knees J Rheumatol 2002 29 3 570 574 11908575

[203] Mathes T Klaßen P Pieper D Frequency of data extraction errors and methods to increase data extraction quality: a methodological review BMC Med Res Methodol 2017 17 1 152 10.1186/s12874-017-0431-4 29179685 PMC5704562

[204] Buckley CD Ospelt C Gay S Midwood KS Location, location, location: how the tissue microenvironment affects inflammation in RA Nat Rev Rheumatol 2021 17 4 195 212 10.1038/s41584-020-00570-2 33526927

[205] Sharma L Osteoarthritis of the knee N Engl J Med 2021 384 1 51 59 10.1056/NEJMcp1903768 33406330

